# Supplementary material for: The Evolution and Characterization of the RNA Interference Pathways in Lophotrochozoa
Source: Genome Biol Evol. 2024 May 7;16(5):evae098. doi: 10.1093/gbe/evae098 (PMC11114477; doi:10.1093/gbe/evae098)
Supplement: evae098_Supplementary_Data [file evae098_supplementary_data.zip › Supplementary_materials.zip!/selected_miRNAs/NoGe_selected_miRNAs/mirdeep_pdf_files/NMRB01003818.1_Notospermus_geniculatus_strain_Ushimado_scaffold3818,_whole_genome_shotgun_sequence_44606.pdf]

Provisional ID : NMRB01003818.1_Notospermus_geniculatus_strain_Ushimado_scaffold3818,_whole_genome_shotgun_sequence_44606
Score total : 46767.3
Score for star read(s) : 3.9
Score for read counts : 46758.7
Score for mfe : 1.6
Score for randfold : 0
Score for cons. seed : 3
Total read count : 91727
Mature read count : 91624
Loop read count : 5
Star read count : 98

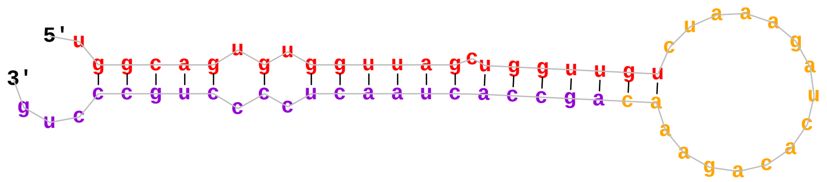

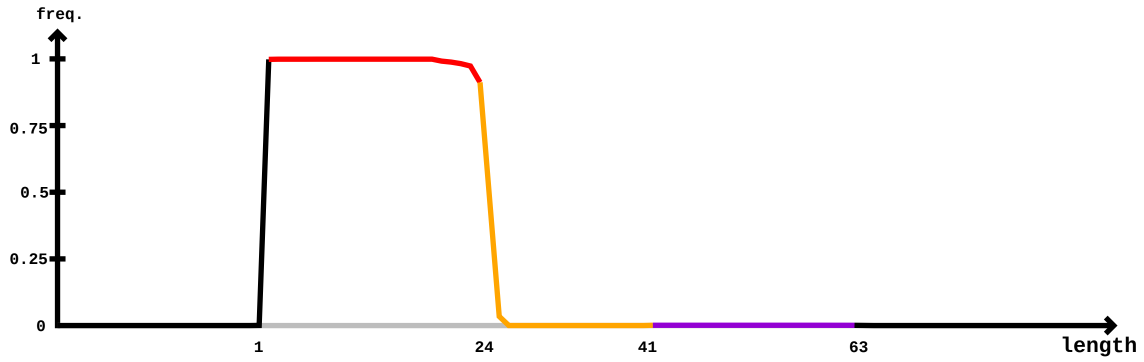


```
                              Mature                     Star
5'- aaauaaacccguguuguuucuuggcagugugguuagcugguugucuaaagaucacagaaacagccacuaacuccccugcccuggaaagacgcggaauaacucgagugcc   -3'   obs
    aaauaaacccguguuguuucuuggcagugugguuagcugguugucuaaagaucacagaaacagccacuaacuccccugcccuggaaagacgcggaauaacucgagugcc         exp
    .......(((((((.((((..(((((.(.((((((.(((((((...............))))))))))))).).))))).)))).)))))))...............    reads   mm   sample
    .....................uggcagugugguuagcugU....................................................................    4       1    Fm2
    .....................uggcagugugguuagcuggG...................................................................    1       1    Fm2
    .....................uggcagugugguuagcuggu...................................................................    3       0    Fm2
    .....................uggcagugugguuagcugguu..................................................................    10      0    Fm2
    .....................uggcagugugguuagcuggGu..................................................................    1       1    Fm2
    .....................Gggcagugugguuagcugguug.................................................................    1       1    Fm2
    .....................uggcaNugugguuagcugguug.................................................................    1       1    Fm2
    .....................uggcagugugguuagcugguGg.................................................................    1       1    Fm2
    .....................uggcagugugguuagcugguuU.................................................................    16      1    Fm2
    .....................uggcCgugugguuagcugguug.................................................................    1       1    Fm2
    .....................uggcagugugguuagcAgguug.................................................................    1       1    Fm2
    .....................uggcagugugguuagcugguuA.................................................................    4       1    Fm2
    .....................uggcagugugAuuagcugguug.................................................................    1       1    Fm2
    .....................uggcagugugguuagcugguug.................................................................    55      0    Fm2
    .....................uggcagugugguuUgcugguugu................................................................    1       1    Fm2
    .....................uAgcagugugguuagcugguugu................................................................    1       1    Fm2
    .....................uggcagugugUuuagcugguugu................................................................    1       1    Fm2
    .....................uggcaguguAguuagcugguugu................................................................    1       1    Fm2
    .....................uggcagugugguCagcugguugu................................................................    1       1    Fm2
    .....................Cggcagugugguuagcugguugu................................................................    1       1    Fm2
    .....................uggcagugugguuagUugguugu................................................................    1       1    Fm2
    .....................uggcagugugAuagcugguugu.................................................................    1       1    Fm2
    .....................uggcagugugguuagcugguugG................................................................    12      1    Fm2
    .....................ugCcagugugguuagcugguugu................................................................    1       1    Fm2
    .....................uggcagugugCuuagcugguugu................................................................    3       1    Fm2
    .....................uggcagugAgguuagcugguugu................................................................    1       1    Fm2
    .....................uggcaNugugguuagcugguugu................................................................    1       1    Fm2
    .....................Nggcagugugguuagcugguugu................................................................    2       1    Fm2
    .....................uggcagugugguuagcugguugA................................................................    7       1    Fm2
    .....................uggAagugugguuagcugguugu................................................................    1       1    Fm2
    .....................uggcCgugugguuagcugguugu................................................................    1       1    Fm2
    .....................uggcagugugguuagcAgguugu................................................................    4       1    Fm2
    .....................uggcagugugAuuagcugguugu................................................................    3       1    Fm2
```

```
                    Mature                                  Star
aaauaacccguguuguuucuuggcagugugguuagcugguugucuaaagaucacagaaacagccacuaacuccccugcccuggaaagacgcggaauaacucgagugcc
....................uggcagugugguuagcuUguugu.................................................................   7     1   Fm2
....................uggcagugugguuagcugguugu.................................................................   2110  0   Fm2
....................uggcaguCugguuagcugguugu.................................................................   1     1   Fm2
....................uggcagugugguuagcGgguugu.................................................................   4     1   Fm2
....................uggcagugugguuagcugguuUu.................................................................   1     1   Fm2
....................uggGagugugguuagcugguugu.................................................................   1     1   Fm2
....................uggcagugugguuagcCgguugu.................................................................   6     1   Fm2
....................uggcagugugguuagcuAguugu.................................................................   7     1   Fm2
....................uggcagugugguuagcuCguugu.................................................................   15    1   Fm2
....................uggcagugugguuagcugguuguc................................................................   1     0   Fm2
....................uggcagugugguuagcugguuguA................................................................   65    1   Fm2
....................uggcagugugguuagcugguuguG................................................................   1     1   Fm2
....................uggcagugugguuagcugguuguU................................................................   444   1   Fm2
....................uggcagugugguuagcugguuguUu...............................................................   211   1   Fm2
....................uggcagugugguuagcugguuguAu...............................................................   22    1   Fm2
.........................gugugguuagcugguugu.................................................................   1     0   Fm2
...........................................cuaaagaucacagaaacU...............................................   5     1   Fm2

....................uggcagugugguuagcug......................................................................   1     0   Fm1
....................uggcagugugguuagUug......................................................................   1     1   Fm1
....................uggcagugugguuagcugg.....................................................................   2     0   Fm1
....................uggcagugugguuagcuAgu....................................................................   1     1   Fm1
....................uggcagugugguuagcuggu....................................................................   4     0   Fm1
....................uggcagugugCuuagcuggu....................................................................   1     1   Fm1
....................uggcagugugguuagcugguu...................................................................   14    0   Fm1
....................uggcagugugguuagcugguGg..................................................................   1     1   Fm1
....................uggcagugugguuagcugguug..................................................................   122   0   Fm1
....................uggcagugugAuuagcugguug..................................................................   2     1   Fm1
....................uggcCgugugguuagcugguug..................................................................   1     1   Fm1
....................uggcagugugguuagcugguuU..................................................................   13    1   Fm1
....................uggcagugugguuagcugguugG.................................................................   4     1   Fm1
....................uggcagugugguuagcuAguugu.................................................................   9     1   Fm1
....................uggcagugugguuagcCgguugu.................................................................   8     1   Fm1
....................uggcaguguUguuagcugguugu.................................................................   2     1   Fm1
....................uggcagugugguuagcuggGugu.................................................................   1     1   Fm1
....................uggcagugugguuagcugguugA.................................................................   14    1   Fm1
....................uggcagugugguuagcuCguugu.................................................................   9     1   Fm1
....................uggcaguguggAuagcugguugu.................................................................   1     1   Fm1
....................uggcagugugguuagcugguugC.................................................................   1     1   Fm1
....................Nggcagugugguuagcugguugu.................................................................   1     1   Fm1
....................uggcagugugguuagcugguGgu.................................................................   1     1   Fm1
....................uggcagugugAuuagcugguugu.................................................................   2     1   Fm1
....................uggcagugugguuagcugguuUu.................................................................   9     1   Fm1
....................uggcGgugugguuagcugguugu.................................................................   1     1   Fm1
....................uggcagugugguuagcuUguugu.................................................................   6     1   Fm1
....................uggcagugugguuagcAgguugu.................................................................   2     1   Fm1
....................uggcagugugUuuagcugguugu.................................................................   1     1   Fm1
....................uggcagugugguuagcugguugu.................................................................   1348  0   Fm1
....................uAgcagugugguuagcugguugu.................................................................   1     1   Fm1
....................uggcagugugCuuagcugguugu.................................................................   3     1   Fm1
....................uggcagugugguuagcugguuguU................................................................   247   1   Fm1
....................uggcagugugguuagcugguuguA................................................................   23    1   Fm1
....................uggcagugugguuagcuAguugucu...............................................................   1     1   Fm1
....................uggcagugugguuagcugguuguGu...............................................................   2     1   Fm1
....................uggcagugugguuagcugguuguUu...............................................................   123   1   Fm1
....................uggcagugugguuagcugguugucu...............................................................   1     0   Fm1
....................uggcagugugguuagcugguuguAu...............................................................   6     1   Fm1
.....................ggcagugugguuagcugguuguU................................................................   3     1   Fm1
............................................................agccacuaacuccccugcc.............................   2     0   Fm1
............................................................agccacuaacuccccugcccu...........................   2     0   Fm1
............................................................Ugccacuaacuccccugcccug..........................   1     1   Fm1
............................................................agccacuaacuccccugcccug..........................   7     0   Fm1
............................................................agccacuaacuccccugcccugA.........................   5     1   Fm1

...................Cuggcagugugguuagcugguug..................................................................   9     1   Mm1
...................Cuggcagugugguuagcugguugu.................................................................   7     1   Mm1
...................uuggcagugugguuagcugguuguU................................................................   1     1   Mm1
....................uggcagugugguuagcAg......................................................................   1     1   Mm1
....................uggcagugugguuagcug......................................................................   18    0   Mm1
....................uggcagugugguuagcugg.....................................................................   35    0   Mm1
```

```
                    Mature                                  Star
aaauaacccguguuguuucuuggcagugugguuagcugguugucuaaagaucacagaaacagccacuaacuccccugcccuggaaagacgcggaauaacucgagugcc
....................uggcagugugguuagcugU.....................................................................     9       1    Mm1
....................uggcagugugguuagcuUgu....................................................................     1       1    Mm1
....................uggcagugugguuagcuggu....................................................................     116     0    Mm1
....................uggcagugugAuuagcuggu....................................................................     2       1    Mm1
....................uggcagugugguuagcGggu....................................................................     1       1    Mm1
....................uggcagugugguuagcugUu....................................................................     3       1    Mm1
....................uggcagugugguuagcuAguu...................................................................     1       1    Mm1
....................uggcagugugguuagcAgguu...................................................................     1       1    Mm1
....................uggcagugugguuagcuCguu...................................................................     4       1    Mm1
....................uggcagugugguuagcugguu...................................................................     118     0    Mm1
....................uggcagugugAuuagcugguu...................................................................     1       1    Mm1
....................uggcagugugguuagcugUuu...................................................................     5       1    Mm1
....................uggcagugugCuuagcugguug..................................................................     1       1    Mm1
....................uggcagugugguuagcugguCg..................................................................     1       1    Mm1
....................uggcagugugguuagcugguuU..................................................................     68      1    Mm1
....................uggcagugugguuagUugguug..................................................................     1       1    Mm1
....................uggcagugugguuagcugguAg..................................................................     1       1    Mm1
....................uggcagugugguuagcuCguug..................................................................     7       1    Mm1
....................uggcagugugAuuagcugguug..................................................................     2       1    Mm1
....................uggcagugugguuagcAgguug..................................................................     4       1    Mm1
....................uggcagugugguuagcCgguug..................................................................     2       1    Mm1
....................uggcagugugguuagcGgguug..................................................................     3       1    Mm1
....................uggcagugugguuagcuAguug..................................................................     5       1    Mm1
....................uggGagugugguuagcugguug..................................................................     1       1    Mm1
....................uggcagugugguuagcugguug..................................................................     1342    0    Mm1
....................uggcagugugguuagcugUuug..................................................................     1       1    Mm1
....................uggcagugAgguuagcugguug..................................................................     1       1    Mm1
....................uggcagugugguuagcuUguug..................................................................     3       1    Mm1
....................uggAagugugguuagcugguug..................................................................     1       1    Mm1
....................uggcUgugugguuagcugguug..................................................................     1       1    Mm1
....................uggcCgugugguuagcugguugu.................................................................     37      1    Mm1
....................Gggcagugugguuagcugguugu.................................................................     1       1    Mm1
....................Cggcagugugguuagcugguugu.................................................................     2       1    Mm1
....................uggcaguguAguuagcugguugu.................................................................     5       1    Mm1
....................uggcagugCgguuagcugguugu.................................................................     7       1    Mm1
....................uggcagugugguuaAcugguugu.................................................................     6       1    Mm1
....................uggcagugugguuagcugguAgu.................................................................     2       1    Mm1
....................uggAagugugguuagcugguugu.................................................................     20      1    Mm1
....................uggcaCugugguuagcugguugu.................................................................     1       1    Mm1
....................uggcagugugUuuagcugguugu.................................................................     44      1    Mm1
....................uggcagNgugguuagcugguugu.................................................................     2       1    Mm1
....................uggcaNugugguuagcugguugu.................................................................     7       1    Mm1
....................uggGagugugguuagcugguugu.................................................................     1       1    Mm1
....................uggcagGgugguuagcugguugu.................................................................     4       1    Mm1
....................uggcagugugguuagcuCguugu.................................................................     174     1    Mm1
....................uggcagugugguCagcugguugu.................................................................     5       1    Mm1
....................ugAcagugugguuagcugguugu.................................................................     10      1    Mm1
....................uggcagugugguuagcugAuugu.................................................................     3       1    Mm1
....................uggcaguCugguuagcugguugu.................................................................     3       1    Mm1
....................uggcagugugguGagcugguugu.................................................................     2       1    Mm1
....................uggcagugugguuagcugguugu.................................................................     37991   0    Mm1
....................uggcagugAgguuagcugguugu.................................................................     5       1    Mm1
....................uggcagugugguuagcuggGugu.................................................................     3       1    Mm1
....................uggcagugugguuCgcugguugu.................................................................     2       1    Mm1
....................uggcagugugguuGgcugguugu.................................................................     5       1    Mm1
....................uggcagugugguuagcuggCugu.................................................................     7       1    Mm1
....................uggcUgugugguuagcugguugu.................................................................     7       1    Mm1
....................uAgcagugugguuagcugguugu.................................................................     10      1    Mm1
....................uggcagugugguuagcugCuugu.................................................................     2       1    Mm1
....................uNgcagugugguuagcugguugu.................................................................     2       1    Mm1
....................uggcagugugguuagcugguugA.................................................................     113     1    Mm1
....................ugNcagugugguuagcugguugu.................................................................     1       1    Mm1
....................uggcagugugguuaUcugguugu.................................................................     4       1    Mm1
....................uggcagugugguuagcugguugG.................................................................     37      1    Mm1
....................ugCcagugugguuagcugguugu.................................................................     6       1    Mm1
....................uggcagugugguuagcugguuUu.................................................................     71      1    Mm1
....................uggcagugugguuagcuAguugu.................................................................     201     1    Mm1
....................uggcagugugCuuagcugguugu.................................................................     37      1    Mm1
....................uggcagugugguuagcuUguugu.................................................................     163     1    Mm1
....................uggcaguguggAuagcugguugu.................................................................     3       1    Mm1
```

```
                    Mature                                  Star
aaauaacccguguuguuucuuggcagugugguuagcugguuguCuaaagaucacagaaacagccacuaacuccccugcccuggaaagacgcggaauaacucgagugcc
....................uggcaguguCguuagcugguugu.................................................................  1     1  Mm1
....................uggcagAgugguuagcugguugu.................................................................  2     1  Mm1
....................uggcagugugguuUgcugguugu.................................................................  7     1  Mm1
....................uUgcagugugguuagcugguugu.................................................................  12    1  Mm1
....................uggcaguAugguuagcugguugu.................................................................  4     1  Mm1
....................uggcaUugugguuagcugguugu.................................................................  4     1  Mm1
....................uggUagugugguuagcugguugu.................................................................  7     1  Mm1
....................uggcagugugguuagcGgguugu.................................................................  90    1  Mm1
....................uggcagugugguuagcAgguugu.................................................................  96    1  Mm1
....................Nggcagugugguuagcugguugu.................................................................  19    1  Mm1
....................uggcaAugugguuagcugguugu.................................................................  1     1  Mm1
....................Aggcagugugguuagcugguugu.................................................................  3     1  Mm1
....................uggcagugugguuagcugguugC.................................................................  15    1  Mm1
....................uggcagugugguuagcCgguugu.................................................................  113   1  Mm1
....................uggcagugugguuagcNgguugu.................................................................  1     1  Mm1
....................uggcagugugguuagcuggAugu.................................................................  2     1  Mm1
....................uggcagugugguuagcugguGgu.................................................................  1     1  Mm1
....................uggcaguguggCuagcugguugu.................................................................  6     1  Mm1
....................uggcagugugAuuagcugguugu.................................................................  77    1  Mm1
....................uggcagCgugguuagcugguugu.................................................................  5     1  Mm1
....................uggcagugugguuagUugguugu.................................................................  5     1  Mm1
....................uggcagugGgguuagcugguugu.................................................................  2     1  Mm1
....................uggcagugugguuagAugguugu.................................................................  2     1  Mm1
....................ugUcagugugguuagcugguugu.................................................................  8     1  Mm1
....................uggcagugugguuagcugguuAu.................................................................  1     1  Mm1
....................uCgcagugugguuagcugguugu.................................................................  4     1  Mm1
....................uggcaguguUguuagcugguugu.................................................................  10    1  Mm1
....................uggcGgugugguuagcugguugu.................................................................  13    1  Mm1
....................uggcagugugguuagcugUuugu.................................................................  6     1  Mm1
....................uggcagugugguuagcugguCgu.................................................................  13    1  Mm1
....................uggcagugugguuagcugguuguU................................................................  3613  1  Mm1
....................uggcagugugguuagcugguuguA................................................................  260   1  Mm1
....................uggcagugugguuagcugguuguG................................................................  45    1  Mm1
....................uggcagugugguuagcugguuguc................................................................  9     0  Mm1
....................uggcagugugguuagcugguuguAu...............................................................  168   1  Mm1
....................uggcagugugguuagcugguuguGu...............................................................  1     1  Mm1
....................uggcagugugguuagcugguuguUu...............................................................  1388  1  Mm1
....................uggcagugugguuagcugguugucu...............................................................  33    0  Mm1
.....................ggcagugugguuagcugguugu.................................................................  21    0  Mm1
.....................ggcagugugUuuagcugguugu.................................................................  1     1  Mm1
.....................Ugcagugugguuagcugguugu.................................................................  6     1  Mm1
.....................ggcagugugguuagcugguuguU................................................................  7     1  Mm1
............................................................agccacuaacGccccugcccug..........................  1     1  Mm1
............................................................agccacuaacuccccugcccug..........................  9     0  Mm1
............................................................agccacuaacuccccugcccuU..........................  4     1  Mm1
............................................................agccacuaacuccccugcccugU.........................  7     1  Mm1

..................cuuggcagugugguuagcugguu...................................................................  1     0  Mm2
...................uuggcagugugguuagcugguug..................................................................  2     0  Mm2
...................uuggcagugugguuagcugguuU..................................................................  2     1  Mm2
...................uuggcagugugguuagcugguugG.................................................................  1     1  Mm2
...................uuggcagugugguuagcugguugu.................................................................  3     0  Mm2
....................uggcagugugguuagcug......................................................................  13    0  Mm2
....................uggcagugugguuagcugg.....................................................................  7     0  Mm2
....................uAgcagugugguuagcugg.....................................................................  1     1  Mm2
....................uggcagugugguuagcuggA....................................................................  2     1  Mm2
....................uggcagugugCuuagcuggu....................................................................  1     1  Mm2
....................uggcagugugguuagcuggu....................................................................  13    0  Mm2
....................uggcagugugguuagcugguA...................................................................  1     1  Mm2
....................uggcagugugguuagcuAguu...................................................................  1     1  Mm2
....................uggcagugugguuagcugguu...................................................................  39    0  Mm2
....................uggcagugugguuagcugguuU..................................................................  18    1  Mm2
....................uggcagugugguuagcuCguug..................................................................  1     1  Mm2
....................uggcagugugguuagcugguug..................................................................  255   0  Mm2
....................uggcagugugguuagcAgguug..................................................................  1     1  Mm2
....................uggcagugugguuagcugguugG.................................................................  14    1  Mm2
....................ugUcagugugguuagcugguugu.................................................................  1     1  Mm2
....................uggcCgugugguuagcugguugu.................................................................  2     1  Mm2
....................uggcagugugguuagcuUguugu.................................................................  10    1  Mm2
....................uggUagugugguuagcugguugu.................................................................  2     1  Mm2
```

```
                    Mature                 Star
aaauaacccguguuguuucuuggcagugugguuagcugguugucuaaagaucacagaaacagccacuaacuccccugcccuggaaagacgcggaauaacucgagugcc
....................uggcagugugguuagAugguugu.................................................................   1      1     Mm2
....................uggcagugugguuagcuggCugu.................................................................   1      1     Mm2
....................uggcagugugguuagcugguugu.................................................................   3972   0     Mm2
....................uggcaguUugguuagcugguugu.................................................................   1      1     Mm2
....................uggcagugugguAagcugguugu.................................................................   1      1     Mm2
....................uggcagugugguuagcuAguugu.................................................................   21     1     Mm2
....................uggcagugugguuagcugguugA.................................................................   28     1     Mm2
....................uggAagugugguuagcugguugu.................................................................   1      1     Mm2
....................uggcagugugguuagcGgguugu.................................................................   9      1     Mm2
....................uggcagugugguuagcAgguugu.................................................................   12     1     Mm2
....................uggcaguguggAuagcugguugu.................................................................   1      1     Mm2
....................uAgcagugugguuagcugguugu.................................................................   1      1     Mm2
....................uggcagugugguuCgcugguugu.................................................................   1      1     Mm2
....................uggcagugugguuagcCgguugu.................................................................   11     1     Mm2
....................uggcagugugAuuagcugguugu.................................................................   13     1     Mm2
....................uggcagugugUuuagcugguugu.................................................................   3      1     Mm2
....................uggcagugugguuagUugguugu.................................................................   2      1     Mm2
....................uggcagugugguuaUcugguugu.................................................................   1      1     Mm2
....................uggcaguguggCuagcugguugu.................................................................   2      1     Mm2
....................ugAcagugugguuagcugguugu.................................................................   1      1     Mm2
....................uggcagugugguuagcugguuUu.................................................................   11     1     Mm2
....................Nggcagugugguuagcugguugu.................................................................   1      1     Mm2
....................uggcagugugguuagcugUuugu.................................................................   1      1     Mm2
....................uggcaNugugguuagcugguugu.................................................................   1      1     Mm2
....................uggcagNgugguuagcugguugu.................................................................   1      1     Mm2
....................uUgcagugugguuagcugguugu.................................................................   1      1     Mm2
....................uggcaguguUguuagcugguugu.................................................................   1      1     Mm2
....................uggcagugugguuagcugAuugu.................................................................   2      1     Mm2
....................uggcagugugguuagcuggGugu.................................................................   1      1     Mm2
....................ugCcagugugguuagcugguugu.................................................................   2      1     Mm2
....................uggcagugugguuagcugguugC.................................................................   1      1     Mm2
....................uggcagugCgguuagcugguugu.................................................................   1      1     Mm2
....................uggcaguCugguuagcugguugu.................................................................   1      1     Mm2
....................uggcagugugguuagcuggAugu.................................................................   1      1     Mm2
....................uggcagugugguuagcuCguugu.................................................................   21     1     Mm2
....................uggcagugugCuuagcugguugu.................................................................   5      1     Mm2
....................uggcagugugguuagcugguuAu.................................................................   1      1     Mm2
....................uggcGgugugguuagcugguugu.................................................................   3      1     Mm2
....................uggcagugugguuagcugguAgu.................................................................   1      1     Mm2
....................uggcagugugguuagcugguuguU................................................................   363    1     Mm2
....................uggcagugugguuagcugguuguA................................................................   28     1     Mm2
....................uggcagugugguuagcugguuguG................................................................   1      1     Mm2
....................uggcagugugguuagcugguuguc................................................................   1      0     Mm2
....................uggcagugugguuagcugguugucu...............................................................   4      0     Mm2
....................uggcagugugguuagcugguuguAu...............................................................   19     1     Mm2
....................uggcagugugguuagcugguuguUu...............................................................   143    1     Mm2
....................uggcagugugguuagcugguuguGu...............................................................   1      1     Mm2
....................uggcagugugguuagcugguugucuaa.............................................................   1      0     Mm2
.....................ggcagugugguuagcugguugu.................................................................   1      0     Mm2
.....................ggcagugugguuagcugguuguUu...............................................................   1      1     Mm2
.........................gugugguuagcugguugu.................................................................   2      0     Mm2
............................................................agccacuaacuccccugcccug..........................   5      0     Mm2
............................................................agccacuaacuccccugcccugU.........................   2      1     Mm2

...................uuggcagugugguuagcugg.....................................................................   1      0     Fm3
...................uuggcagugugguuagcugguuA..................................................................   2      1     Fm3
...................uuggcagugugguuagcugguugu.................................................................   8      0     Fm3
....................uggcagugugguuagcug......................................................................   603    0     Fm3
....................uggcagugugguuagcCg......................................................................   3      1     Fm3
....................uggcagugugguuagcAg......................................................................   2      1     Fm3
....................Nggcagugugguuagcug......................................................................   1      1     Fm3
....................uggcagugugguuagcuC......................................................................   2      1     Fm3
....................uggcagugugguAagcug......................................................................   1      1     Fm3
....................uggcagugugUuuagcug......................................................................   2      1     Fm3
....................uggcagugugguuagcuU......................................................................   3      1     Fm3
....................uggAagugugguuagcug......................................................................   3      1     Fm3
....................uggcagugugguuagcuA......................................................................   3      1     Fm3
....................uggcagugugAuuagcug......................................................................   2      1     Fm3
....................uggcagugugguuagcGg......................................................................   1      1     Fm3
....................Gggcagugugguuagcugg.....................................................................   1      1     Fm3
```

```
                    Mature                                      Star
aaauaacccguguuguuucuuggcagugugguuagcugguugucuaaagaucacagaaacagccacuaacuccccugcccuggaaagacgcggaauaacucgagugcc
....................uggcagugugguuagcugA.....................................................................    8      1     Fm3
....................uggcagugugCuuagcugg.....................................................................    2      1     Fm3
....................uggcagugugguuagcuAg.....................................................................    1      1     Fm3
....................uggcagugugguuagcugg.....................................................................    235    0     Fm3
....................uggcagugugguuagcAgg.....................................................................    2      1     Fm3
....................uggcagugugguuagcuCg.....................................................................    2      1     Fm3
....................uggcagugugguuagcugU.....................................................................    10     1     Fm3
....................uggcagugugguuagcGgg.....................................................................    1      1     Fm3
....................uggcagugCgguuagcuggu....................................................................    1      1     Fm3
....................uggcagugGgguuagcuggu....................................................................    1      1     Fm3
....................uggcagugugCuuagcuggu....................................................................    1      1     Fm3
....................uggAagugugguuagcuggu....................................................................    1      1     Fm3
....................uggcagugugguuagcGggu....................................................................    1      1     Fm3
....................uggcagugugguuagcuggA....................................................................    16     1     Fm3
....................uggcagugugAuuagcuggu....................................................................    1      1     Fm3
....................uggcagugugguuagcugUu....................................................................    10     1     Fm3
....................uggcagugugguuagcuggu....................................................................    291    0     Fm3
....................uggcagugugguuagcuUgu....................................................................    2      1     Fm3
....................uggcagugugguuagcuAgu....................................................................    1      1     Fm3
....................uggcGgugugguuagcuggu....................................................................    1      1     Fm3
....................uggcagGgugguuagcugguu...................................................................    1      1     Fm3
....................uggcagugugguuagcuUguu...................................................................    2      1     Fm3
....................uggcagugugguuagcugguu...................................................................    520    0     Fm3
....................uggcagugugguuagcAgguu...................................................................    1      1     Fm3
....................uggcagugugguuaUcugguu...................................................................    1      1     Fm3
....................ugUcagugugguuagcugguu...................................................................    2      1     Fm3
....................uggcagugugguuagAugguu...................................................................    1      1     Fm3
....................uggcagugugguuagcugUuu...................................................................    1      1     Fm3
....................uggcagugugguuagGugguu...................................................................    1      1     Fm3
....................uggcagugugAuuagcugguu...................................................................    1      1     Fm3
....................uggcagugugCuuagcugguu...................................................................    1      1     Fm3
....................uggcagugugguuagcGgguu...................................................................    1      1     Fm3
....................uggcagugugguuUgcugguu...................................................................    1      1     Fm3
....................uggcagugugguuagcCgguu...................................................................    1      1     Fm3
....................uggcagugugguuagcuCguu...................................................................    4      1     Fm3
....................uggcaguguUguuagcugguug..................................................................    1      1     Fm3
....................uggcagugugUuuagcugguug..................................................................    3      1     Fm3
....................uggcagugugguuagcGgguug..................................................................    9      1     Fm3
....................uggcagugugguuagcuCguug..................................................................    20     1     Fm3
....................uggcagugugguuagcuAguug..................................................................    20     1     Fm3
....................uAgcagugugguuagcugguug..................................................................    2      1     Fm3
....................uggcagugugguuagUugguug..................................................................    15     1     Fm3
....................uggcaguguCguuagcugguug..................................................................    1      1     Fm3
....................uggcaUugugguuagcugguug..................................................................    1      1     Fm3
....................uggAagugugguuagcugguug..................................................................    2      1     Fm3
....................ugUcagugugguuagcugguug..................................................................    1      1     Fm3
....................uggcCgugugguuagcugguug..................................................................    2      1     Fm3
....................uggcagugugguuagcuUguug..................................................................    18     1     Fm3
....................uggcaguAugguuagcugguug..................................................................    1      1     Fm3
....................uggcaguCugguuagcugguug..................................................................    1      1     Fm3
....................uggcagugugguuagcugguuU..................................................................    72     1     Fm3
....................uggcagugugguuagcugguug..................................................................    3115   0     Fm3
....................uggcagugugguuagcugguCg..................................................................    1      1     Fm3
....................uggcagugugguuagcAgguug..................................................................    11     1     Fm3
....................uCgcagugugguuagcugguug..................................................................    1      1     Fm3
....................uggcagugugCuuagcugguug..................................................................    1      1     Fm3
....................uggcagugugAuuagcugguug..................................................................    4      1     Fm3
....................uggcagugugguuagcugAuug..................................................................    1      1     Fm3
....................ugCcagugugguuagcugguug..................................................................    1      1     Fm3
....................uggcagugugguuagcCgguug..................................................................    10     1     Fm3
....................ugAcagugugguuagcugguug..................................................................    1      1     Fm3
....................uggGagugugguuagcugguug..................................................................    1      1     Fm3
....................uggcagugugguuagcuggAug..................................................................    2      1     Fm3
....................uggUagugugguuagcugguug..................................................................    1      1     Fm3
....................uggcUgugugguuagcugguug..................................................................    1      1     Fm3
....................uggcagugugguuagcugguuA..................................................................    4      1     Fm3
....................uggcagugugguuGgcugguug..................................................................    1      1     Fm3
....................Cggcagugugguuagcugguug..................................................................    2      1     Fm3
....................uggcagugugguuagcuggGugu.................................................................    1      1     Fm3
....................uggcagugugguuagcuggCugu.................................................................    2      1     Fm3
```

Mature   Star

```
aaauaacccguguuguuucuuggcagugugguuagcugguugucuaaagaucacagaaacagccacuaacuccccugcccuggaaagacgcggaauaacucgagugcc
....................uggcagNgugguuagcugguugu.................................................................  1      1  Fm3
....................uggcagugugguuagcugguugG.................................................................  25     1  Fm3
....................uggcagugugguuagcugguugC.................................................................  5      1  Fm3
....................uggcaNugugguuagcugguugu.................................................................  3      1  Fm3
....................uggcCgugugguuagcugguugu.................................................................  5      1  Fm3
....................uCgcagugugguuagcugguugu.................................................................  4      1  Fm3
....................uggUagugugguuagcugguugu.................................................................  3      1  Fm3
....................uggGagugugguuagcugguugu.................................................................  3      1  Fm3
....................ugUcagugugguuagcugguugu.................................................................  7      1  Fm3
....................uggcagugugguuagAugguugu.................................................................  1      1  Fm3
....................Aggcagugugguuagcugguugu.................................................................  4      1  Fm3
....................uggcagugugguuagcuUguugu.................................................................  75     1  Fm3
....................uggcagAgugguuagcugguugu.................................................................  3      1  Fm3
....................uggcagugugguuagcCgguugu.................................................................  67     1  Fm3
....................uggcagugAgguuagcugguugu.................................................................  3      1  Fm3
....................Nggcagugugguuagcugguugu.................................................................  8      1  Fm3
....................uggcagugugguuagUugguugu.................................................................  25     1  Fm3
....................uggcagugugguuagcugguuUu.................................................................  8      1  Fm3
....................uggcagugugguuagcugguugA.................................................................  453    1  Fm3
....................uggcagugugguuagcuCguugu.................................................................  112    1  Fm3
....................ugAcagugugguuagcugguugu.................................................................  6      1  Fm3
....................uggcUgugugguuagcugguugu.................................................................  1      1  Fm3
....................uggcaguguggGuagcugguugu.................................................................  1      1  Fm3
....................uggcagugugguuagcugguugu.................................................................  18205  0  Fm3
....................Cggcagugugguuagcugguugu.................................................................  1      1  Fm3
....................uggcaAugugguuagcugguugu.................................................................  1      1  Fm3
....................uggcaguguUguuagcugguugu.................................................................  2      1  Fm3
....................ugCcagugugguuagcugguugu.................................................................  1      1  Fm3
....................uggcagugugguuagcugUuugu.................................................................  4      1  Fm3
....................uggcagugugguuagcugguuAu.................................................................  1      1  Fm3
....................uggcaguUugguuagcugguugu.................................................................  1      1  Fm3
....................uggcaguguggCagcugguugu..................................................................  1      1  Fm3
....................uggcagugugguuagcugguAgu.................................................................  1      1  Fm3
....................uggcaguAugguuagcugguugu.................................................................  2      1  Fm3
....................uUgcagugugguuagcugguugu.................................................................  5      1  Fm3
....................uggcagugugguAagcugguugu.................................................................  1      1  Fm3
....................uggcagugugguuagcAgguugu.................................................................  50     1  Fm3
....................uggcagugugguuagcuNguugu.................................................................  1      1  Fm3
....................uggcagGgugguuagcugguugu.................................................................  1      1  Fm3
....................uggcagCgugguuagcugguugu.................................................................  2      1  Fm3
....................uggcagugugguuagcugguuCu.................................................................  1      1  Fm3
....................uggcagugugguuagcuggAugu.................................................................  4      1  Fm3
....................uggcagugugguuagcuAguugu.................................................................  96     1  Fm3
....................uggcaguguAguuagcugguugu.................................................................  2      1  Fm3
....................uggcagugugguuaCcugguugu.................................................................  1      1  Fm3
....................uggcagugugAuuagcugguugu.................................................................  40     1  Fm3
....................uggcagugugguuagcugguGgu.................................................................  4      1  Fm3
....................uggcagugugCuuagcugguugu.................................................................  17     1  Fm3
....................uggcaguCugguuagcugguugu.................................................................  3      1  Fm3
....................uggcagugugguuagGugguugu.................................................................  1      1  Fm3
....................uggcagugugUuuagcugguugu.................................................................  11     1  Fm3
....................uggcagugugguuaAcugguugu.................................................................  1      1  Fm3
....................uggcagugugguuagcGgguugu.................................................................  49     1  Fm3
....................uggAagugugguuagcugguugu.................................................................  9      1  Fm3
....................uggcagugGgguuagcugguugu.................................................................  2      1  Fm3
....................uggcaguguggCuagcugguugu.................................................................  2      1  Fm3
....................uggcGgugugguuagcugguugu.................................................................  3      1  Fm3
....................uggcagugugguuaUcugguugu.................................................................  3      1  Fm3
....................uggcaUugugguuagcugguugu.................................................................  3      1  Fm3
....................uggcagugCgguuagcugguugu.................................................................  4      1  Fm3
....................uggcaguguggAuagcugguugu.................................................................  2      1  Fm3
....................uAgcagugugguuagcugguugu.................................................................  8      1  Fm3
....................uggcagugugguuUgcugguugu.................................................................  2      1  Fm3
....................uggcagugugguuGgcugguugu.................................................................  5      1  Fm3
....................uggcagugugguuagcugguuguA................................................................  133    1  Fm3
....................uggcagugugguuagcugguuguU................................................................  1169   1  Fm3
....................uggcagugugguuagcugguuguc................................................................  9      0  Fm3
....................uggcagugugguuagcugguuguG................................................................  11     1  Fm3
....................uggcagugugguuagcugguuguAu...............................................................  55     1  Fm3
....................uggcagugugguuagcugguugucu...............................................................  10     0  Fm3
```

Mature **Star**

```
aaauaacccguguuguuucuuggcagugugguuagcugguugucuaaagaucacagaaacagccacuaacuccccugcccuggaaagacgcggaauaacucgagugcc
....................uggcagugugguuagcugguuguUu...............................................................   281   1   Fm3
....................uggcagugugguuagcugguuguGu...............................................................   4     1   Fm3
....................uggcagugugguuagcugguugucua..............................................................   2     0   Fm3
.....................ggcagugugguuagcuggu....................................................................   1     0   Fm3
.....................ggcagugugguuagcugguug..................................................................   1     0   Fm3
.....................ggcagugugguuagcugguCg..................................................................   1     1   Fm3
.....................Ugcagugugguuagcugguug..................................................................   1     1   Fm3
.....................Ugcagugugguuagcugguugu.................................................................   2     1   Fm3
.....................ggcagugugguuagcugguugu.................................................................   12    0   Fm3
.....................ggcagugugguuagcuggAugu.................................................................   1     1   Fm3
.....................ggcagugugguuagcugguuUu.................................................................   1     1   Fm3
.....................ggcagAgugguuagcugguugu.................................................................   1     1   Fm3
.....................ggcagugugguuagcugguuguU................................................................   1     1   Fm3
.....................ggcagugugguuagcugguuguUu...............................................................   1     1   Fm3
........................agugugguuagcugguugu.................................................................   1     0   Fm3
...........................................................cagccacuaacuccccugcccu...........................   3     0   Fm3
...........................................................cagccacuaacuccccuAcccu...........................   1     1   Fm3
............................................................aUccacuaacuccccugcccug..........................   1     1   Fm3
............................................................agccacuaacuccccugcccug..........................   25    0   Fm3
............................................................agcAacuaacuccccugcccug..........................   1     1   Fm3
............................................................agccacuaacuccccugcccugU.........................   3     1   Fm3
............................................................agccacuaacuccccugcccugA.........................   1     1   Fm3

...................uuggcagugugguuagcuggu....................................................................   1     0   Mm3
...................uuggcagugugguuagcugguugu.................................................................   3     0   Mm3
...................uuggcagugugguuagcAgguugu.................................................................   2     1   Mm3
...................uuggcagugugguuagcugguuguUu...............................................................   1     1   Mm3
....................uggcagugugguuagcug......................................................................   10    0   Mm3
....................uggcagugugguuagcuA......................................................................   1     1   Mm3
....................uggcagugugguuagcugg.....................................................................   12    0   Mm3
....................uggcagugugguuagcugA.....................................................................   4     1   Mm3
....................uggcagugugguuaAcugg.....................................................................   1     1   Mm3
....................uggcagugugguuagcuAg.....................................................................   1     1   Mm3
....................uggcagugugguuagcugUu....................................................................   3     1   Mm3
....................uggcagugugguuagcuggu....................................................................   41    0   Mm3
....................uggcagugugguuagcuAgu....................................................................   1     1   Mm3
....................uggcagugugguuagcuggA....................................................................   5     1   Mm3
....................uggcagugugguuagcugguu...................................................................   74    0   Mm3
....................Aggcagugugguuagcugguu...................................................................   1     1   Mm3
....................uggcagugugguuagcGgguu...................................................................   1     1   Mm3
....................uggcagugugguuagcuCguu...................................................................   2     1   Mm3
....................uggcagugugguuagcuAguu...................................................................   2     1   Mm3
....................uggcagugugguuagcugguA...................................................................   2     1   Mm3
....................uggcagugugguuagcCgguug..................................................................   1     1   Mm3
....................uggcagCgugguuagcugguug..................................................................   1     1   Mm3
....................uggcagugugguuagcuUguug..................................................................   2     1   Mm3
....................uggcagugugAuuagcugguug..................................................................   1     1   Mm3
....................uggcCgugugguuagcugguug..................................................................   2     1   Mm3
....................uggcagugugUuuagcugguug..................................................................   1     1   Mm3
....................uggcagugugguuagcugguuU..................................................................   44    1   Mm3
....................uggcagugugguuagcGgguug..................................................................   1     1   Mm3
....................uggcagNgugguuagcugguug..................................................................   1     1   Mm3
....................uAgcagugugguuagcugguug..................................................................   1     1   Mm3
....................uggcagugugguuagcuCguug..................................................................   2     1   Mm3
....................uggcagugugguuagcugguug..................................................................   273   0   Mm3
....................uggcagugugguuagcuAguugu.................................................................   30    1   Mm3
....................ugUcagugugguuagcugguugu.................................................................   1     1   Mm3
....................uggcUgugugguuagcugguugu.................................................................   1     1   Mm3
....................uggcaguguggAuagcugguugu.................................................................   1     1   Mm3
....................uggcagugugguuGgcugguugu.................................................................   1     1   Mm3
....................uggcagugugguuCgcugguugu.................................................................   1     1   Mm3
....................ugAcagugugguuagcugguugu.................................................................   1     1   Mm3
....................uggcagugugguuagcugguugA.................................................................   33    1   Mm3
....................uggcagNgugguuagcugguugu.................................................................   1     1   Mm3
....................uggcagugugguuagcugguugG.................................................................   8     1   Mm3
....................uUgcagugugguuagcugguugu.................................................................   2     1   Mm3
....................uggcagugugguuagcugUuugu.................................................................   3     1   Mm3
....................uCgcagugugguuagcugguugu.................................................................   1     1   Mm3
....................uggcaUugugguuagcugguugu.................................................................   1     1   Mm3
....................uggcCgugugguuagcugguugu.................................................................   2     1   Mm3
```

Mature Star

```
aaauaacccguguuguuucuuggcagugugguuagcugguugucuaaaagaucacagaaacagccacuaacuccccugcccuggaaagacgcggaauaacucgagugcc
....................uggcaguguAguuagcugguugu..................................................................	2	1	Mm3
....................uggcagugugguuagcugguuUu..................................................................	19	1	Mm3
....................uggcagugugguuUgcugguugu..................................................................	1	1	Mm3
....................Nggcagugugguuagcugguugu..................................................................	4	1	Mm3
....................uggcagugugUuuagcugguugu..................................................................	3	1	Mm3
....................uggcagugugCuuagcugguugu..................................................................	5	1	Mm3
....................Aggcagugugguuagcugguugu..................................................................	1	1	Mm3
....................uggcagugugAuuagcugguugu..................................................................	9	1	Mm3
....................uggcagAgugguuagcugguugu..................................................................	2	1	Mm3
....................Cggcagugugguuagcugguugu..................................................................	3	1	Mm3
....................uggcagugugguuagcugguugu..................................................................	5743	0	Mm3
....................uggcagugugguuagcuggAugu..................................................................	1	1	Mm3
....................uggcagugugguuagcuUguugu..................................................................	27	1	Mm3
....................uggcagugugguuagcGgguugu..................................................................	11	1	Mm3
....................uggUagugugguuagcugguugu..................................................................	1	1	Mm3
....................uggcagugugguuagAugguugu..................................................................	1	1	Mm3
....................uggcagugugguuagcuCguugu..................................................................	22	1	Mm3
....................uggcagugugguuagcCgguugu..................................................................	11	1	Mm3
....................uggcGgugugguuagcugguugu..................................................................	3	1	Mm3
....................uggcagugugguuagcAgguugu..................................................................	11	1	Mm3
....................uggcagugugguuagcugguuCu..................................................................	1	1	Mm3
....................uggcagugGgguuagcugguugu..................................................................	2	1	Mm3
....................uggcagugugguuagcugguugC..................................................................	2	1	Mm3
....................uAgcagugugguuagcugguugu..................................................................	3	1	Mm3
....................uggcaguAugguuagcugguugu..................................................................	1	1	Mm3
....................uggcagugugguuagcugguuguc.................................................................	4	0	Mm3
....................uggcagugugguuagcugguuguG.................................................................	9	1	Mm3
....................uggcagugugguuagcugguuguA.................................................................	116	1	Mm3
....................uggcagugugguuagcugguuguU.................................................................	1182	1	Mm3
....................uggcagugugguuagcugguugucu................................................................	5	0	Mm3
....................uggcagugugguuagcugguuguUu................................................................	562	1	Mm3
....................uggcagugugguuagcugguuguAu................................................................	99	1	Mm3
....................uggcagugugguuagcugguugucua...............................................................	1	0	Mm3
.....................ggcagugugguuagcuggu.....................................................................	1	0	Mm3
.....................ggcagugugguuagcugguugu..................................................................	4	0	Mm3
.....................ggcagugugguuagcugguuguUu................................................................	1	1	Mm3
.......................cagugugguuagcugguugucu................................................................	1	0	Mm3
............................................uaaagaucacagaaacagccacuaacuc.....................................	1	0	Mm3
............................................................cagccacuaacuccccugcccu...........................	1	0	Mm3
.............................................................agccacuaacuccccugcccu...........................	2	0	Mm3
.............................................................agccacuaacuccccugcccug..........................	13	0	Mm3
.............................................................agccacuaacuccccugcccugU.........................	2	1	Mm3
```
